# Recent Improvement of Medical Optical Fibre Pressure and Temperature Sensors

**DOI:** 10.3390/bios5030432

**Published:** 2015-07-13

**Authors:** Sven Poeggel, Dineshbabu Duraibabu, Kyriacos Kalli, Gabriel Leen, Gerard Dooly, Elfed Lewis, Jimmy Kelly, Maria Munroe

**Affiliations:** 1 Optical Fibre Sensors Research Centre-University of Limerick, Limerick MS1019, Ireland; E-Mails: ddineshbabu@hotmail.com (D.D.); Gabriel.leen@ul.ie (G.L.); gerard.dooly@ul.ie (G.D.); Elfed.Lewis@ul.ie (E.L.); 2 Nanophotonics Research Laboratory, Cyprus University of Technology, Lemesos 3036, Cyprus; E-Mail: kkalli@cytanet.com.cy; 3 University of Limerick, Chemical & Environmental Science, Limerick MS1019, Ireland; E-Mails: James.kelly@ul.ie (J.K.); Maria.Munroe@ul.ie (M.M.)

**Keywords:** optical fibre sensors, Fabry–Perot interferometer, pressure sensors

## Abstract

This investigation describes a detailed analysis of the fabrication and testing of optical fibre pressure and temperature sensors (OFPTS). The optical sensor of this research is based on an extrinsic Fabry–Perot interferometer (EFPI) with integrated fibre Bragg grating (FBG) for simultaneous pressure and temperature measurements. The sensor is fabricated exclusively in glass and with a small diameter of 0.2 mm, making it suitable for volume-restricted bio-medical applications. Diaphragm shrinking techniques based on polishing, hydrofluoric (HF) acid and femtosecond (FS) laser micro-machining are described and analysed. The presented sensors were examined carefully and demonstrated a pressure sensitivity in the range of $s_{p}$ = 2–10 $\frac{\text{nm}}{\text{kPa}}$ and a resolution of better than ΔP = 10 Pa (0.1 cm H${}_{2}$O). A static pressure test in 38 cmH${}_{2}$O shows no drift of the sensor in a six-day period. Additionally, a dynamic pressure analysis demonstrated that the OFPTS never exceeded a drift of more than 130 Pa (1.3 cm H${}_{2}$O) in a 12-h measurement, carried out in a cardiovascular simulator. The temperature sensitivity is given by k=10.7 $\frac{\text{pm}}{\text{K}}$, which results in a temperature resolution of better than ΔT = 0.1 K. Since the temperature sensing element is placed close to the pressure sensing element, the pressure sensor is insensitive to temperature changes.

## 1. Introduction

Sensors in research and industry demand measurements with great accuracy and high stability. Particularly in the medical field, a sensor has to be very accurate and should exhibit a high long-term stability with very little drift. Additionally, medical sensors also need to be small in size, otherwise they can be uncomfortable for the patient and cumbersome for the clinician, particularly when used in restricted volume areas, such as in blood vessels, the lungs or the brain. During an operation, sensors have to be resistant to the application of mechanical force. Medical equipment has to be reliable, also under harsh environmental conditions. Apart from the physical characteristics, the sensor should also have no effect on the measurement environment or on the patient’s well-being.

Recent developments in optical fibre sensors (OFS) demonstrated an increasing growth in medical applications [1] to measure parameters, including pressure [2], force [3], temperature [4] or refractive index [5]. Extrinsic Fabry–Perot interferometer (EFPI) sensors have become an established technology for pressure sensing, finding applications in a number of diverse industrial areas, for example in acoustic engineering [6], downstream oil and gas [7] and structural health monitoring (SHM) [8]. However, in the case of the medical field, OFS exhibit inherent advantages, such as their small size and the biocompatible glass structure. These sensors are resistant to organic and chemical degradation, which makes them compatible for animal and human biology. Temperature sensors based on fibre Bragg grating (FBG) and Fabry–Perot interferometer (FPI) pressure sensors are well known in research and industry [9,10].

With regards to medical applications, the development of innovative fibre optic EFPI sensors has been challenging in order to make them fully biocompatible. Although optical fibres and additional glass components, such as glass capillaries, are biocompatible, in many cases, the FPI sensors are sealed with epoxy or other chemical compounds. As a result, EFPI sensors are often not compliant to the ISO10993 standard for medical *in vivo* applications [11]. Furthermore, a key objective is the improvement of pressure resolution and accuracy over the state-of-the-art sensors. Medical pressure sensors require a typical accuracy of 100 Pa (1 cmH${}_{2}$O).

The OFS presented in this paper is capable of measuring temperature and pressure simultaneously through the combination of an EFPI with an in-built FBG. The simultaneous measurement of pressure and temperature requires only one optical fibre in total, which is advantageous in volume restricted areas. Recent improvements in the fabrication of low-cost optical fibre pressure and temperature sensor (OFPTS) for medical applications are further discussed in this paper. This includes a potentially low-cost fabrication process [12], different etching methods and the combination of the FBG and FPI sensor element in a single fibre. The sensors present in this paper have a pressure resolution of better than ΔP = 10 Pa (0.1 cmH${}_{2}$O) with a temperature resolution of better than ΔT = 0.1 K. Furthermore, since the pressure measurement of an EFPI is affected by temperature, the simultaneous acquisition of both measurements can be used to compensate ambiguity due to cross-sensitivity [13,14,15].

## 2. Theoretical Background

The OFPTS system can be broken down into three parts: (1) the hardware, including broadband light source (BLS) and optical spectrum analyser (OSA); (2) the OFPTS sensing element for parallel pressure and temperature measurements; and (3) a PC with analysis software and tracking algorithms designed for the OFPTS-system. All three parts are designed to achieve a fully bio-compatible, highly sensitive and stable optical sensor system, suited to applications in the medical field.

### 2.1. The Medical Evaluation System

**Figure 1:**
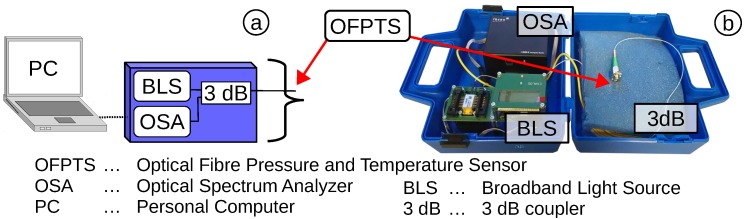
(**a**) Schematic of the optical fibre pressure and temperature sensors (OFPTS) system; (**b**) the hand-case-sized optical fibre system.

The hardware is designed as a prototype system for use in medical evaluations, such as supportive home care diagnostics. The requirements for such a system demand an inexpensive, portable, robust and highly stable system. The optical transmission is based on a BLS (*i.e.*, superluminescent diode (SLED)) with a wavelength range from 1525–1600 nm and a power intensity of 10 dBm. The emitted light travels over a 3-dB coupler to the tip of the OFPTS and back to the OSA (Figure 1a). The OSA, I-MON 512 from Ibsen photonics, has an optical resolution of $0.166\frac{\text{nm}}{\text{pixel}}$, a ±5 pm accuracy and a 997-Hz frame rate. The resolution can be significantly improved using proprietary curve fitting algorithms. Under such circumstances, the resolution is better than 3 pm. The equipment fits into a portable small hand case (18 × 25 × 8 cm), shown in Figure 1b.

### 2.2. Optical Fibre Pressure and Temperature Sensor

**Figure 2:**
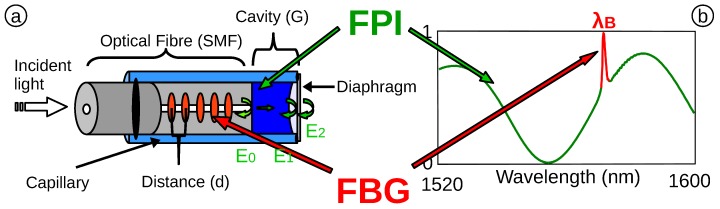
(**a**) Schematic of the optical fibre pressure and temperature sensor; (**b**) reflected optical spectrum of the OFPTS, with Fabry–Perot interferometer (FPI) and FBG.

The sensing element is a combination of FPI and FBG technology, shown schematically in Figure 2a. The continuous FPI spectrum overlaps with a sharp peak at the Bragg wavelength ($\lambda_{B}$) caused by the FBG, shown in Figure 2b. The sensor is constructed entirely from glass in order to guarantee fully bio-compatibility.

#### 2.2.1. Fabry Perot Interferometer (FPI)

The EFPI OFS comprises a Fabry–Perot (FP) cavity structure on the tip of an optical fibre. The FP cavity is formed by three miniature mirrors with low reflectivity of light (${\vec{E}}_{0}$, ${\vec{E}}_{1}$ and ${\vec{E}}_{2}$) that generate a wavelength-selective interference pattern. The intensity (*I*) of the reflected optical spectrum with respect to the light’s wavelength (*λ*) is shown in Equation (1). The cavity of the sensor is completely sealed, which facilitates a bending of the diaphragm (ΔG) when exposed to pressure changes (ΔP). When the distance of the reflecting surface (*G*) or the refractive index ($n_{0}$ or $n_{1}$) is changed, the wavelength responds to the FPI changes accordingly.

(1) $$I=E_{0}\cdot E_{1}\cdot \cos\left(\frac{4\pi \cdot G\cdot n_{0}}{\lambda }\right)+E_{0}\cdot E_{2}\cdot \cos\left(\frac{4\pi \cdot (G\cdot n_{0}+h\cdot n_{1})}{\lambda }\right)+E_{1}\cdot E_{2}\cdot \cos\left(\frac{4\pi \cdot h\cdot n_{1}}{\lambda }\right)$$

Since Young’s modulus (*E*) and Poisson’s ratio (µ) for an all-glass sensor are constant, the high pressure sensitivity $s_{p}$ depends strongly on the diaphragm thickness (*h*) and radius (*r*), shown in Equation (2).

(2) $$s_{p}=\frac{\Delta G}{\Delta P}=\frac{3}{16}\cdot \frac{\left(1-\mu^{2}\right)}{E}\cdot \frac{r^{4}}{h^{3}}$$

For most medical applications, the sensor needs a high sensitivity to achieve a pressure resolution of better than 100 kPa (1 cm H${}_{2}$O). The OFPTS in this research has a single-mode fibre (SMF) demanded inner radius of 65μm. Our recent investigation demonstrated an *in vivo* online pressure measurement in urodynamic analysis with a pressure resolution of 0.1 cm H${}_{2}$O [16]. This resolution is achieved by a pressure sensitivity of $s_{p}\approx 2\frac{\text{nm}}{\text{kPa}}$, based on a 2–3 *μ*m-thick diaphragm. To achieve a highly-sensitive OFPTS, it becomes mandatory to be able to control the shrinking process of the glass diaphragm precisely.

#### 2.2.2. Integrated Fibre Bragg Grating

A FBG is a periodic variation of the refractive index in the core of an SMF [17,18]. The standard method includes a pre-inscription with subsequent FPI fabrication [15,19,20]. Recently, we demonstrated a technique to post inscribe an FBG into an EFPI pressure sensor, using a femtosecond laser (FSL) [21]. The periodical variation results in a selective bandstop filter in the wavelength domain (Equation (3)) with high reflectivity at the Bragg-wavelength $\lambda_{B}=2n_{eff}\cdot \Lambda$, where $n_{eff}$ is the effective refractive index and Λ the grating period. With a change of temperature (ΔT), the distance of the period of the grating in the core changes accordingly. This results in a spectral shift of the Bragg wavelength, determined by the temperature sensitivity (*k*).

(3) $$\Delta \lambda_{B}\left(\Delta T\right)=k\cdot \Delta T$$

The combination of both techniques and the simultaneous measurement allows the construction of a matrix [19], which is based on the differential change of the Bragg wavelength ($\Delta \lambda_{B}$) and the change of the cavity length (ΔG), shown in Equation (4).

(4) $$\left[\begin{array}{c} \Delta \lambda_{B}\\ \Delta G \end{array}\right]=\left[\begin{array}{cc} 0 & k\\ s_{p} & s_{t} \end{array}\right]\left[\begin{array}{c} \Delta P\\ \Delta T \end{array}\right]$$

### 2.3. Software for Medical Evaluation System

**Figure 3:**
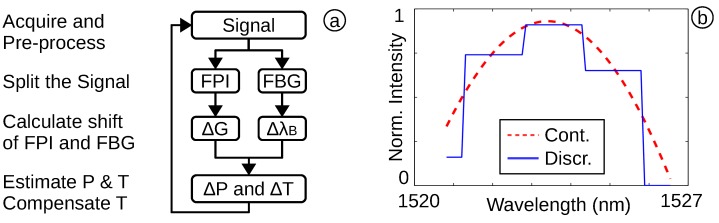
(**a**) The acquired spectrum is split into the FPI and FBG part; (**b**) an acquired FPI spectrum (continuous line) with $1\frac{\text{nm}}{\text{pixel}}$ and the continuous optical spectrum (dashed).

The software of the OFPTS system collects the spectrum, acquired by the OSA, and separates the narrow-band FBG spectrum from the broad-band FPI spectrum. It then independently analyses the shift of both spectra to determine the temperature and pressure change and to compensate for any thermal effects on the FPI sensor. These results are fed back, and in combination with the next frame, they can be used to compensate for noise and bending of the optical fibre. The implemented flow diagram is shown in Figure 3a.

To compensate for the low resolution of an inexpensive and portable interrogator, an advanced algorithm was used. This results in a higher resolution, which can compensate a lower sensitivity and allows for a thicker and less fragile diaphragm. The implemented technique for high pressure resolution is based on an interpolation of the acquired and reprocessed signal. The algorithm calculates the best fit spectrum, based on Equation (1), to achieve a good approximation of the physical parameters. The interpolation (Figure 3b) based on the Levenberg–Marquardt algorithm (LMA) (included in LabVIEW${}^{TM}$ 2012) [2,15,22] and the best fitted parameters are used to calculate the pressure and temperature change.

## 3. Fabrication of an Optical Fibre Pressure and Temperature Sensor (OFPTS)

The conventional fabrication process of the OFPTS can be divided into three stages. First, an FBG is inscribed into the core of an SMF proximately close to the end-face. Afterwards, the SMF with FBG is used to fabricate the EFPI sensor. In the last step, the diaphragm of the EFPI sensor is reduced to a thin membrane to achieve high pressure sensitivity. A new fabrication order with FBG post-inscription is also possible, as recently demonstrated [21].

### 3.1. Fibre Bragg Grating Temperature Sensor

The standard techniques for FBG fabrication are based on using a conventional ultra violet (UV) phase mask inscription method [23] or directly inscribed during SMF fabrication in a specially-designed draw-tower [24]. Newer techniques demonstrated a direct inscription by using an FSL [25].

**Figure 4:**
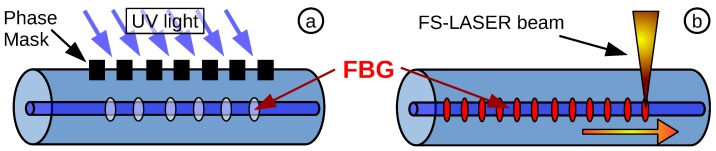
(**a**) FBG inscription into a photosensitive single-mode fibre (SMF) by using a phase mask and UV light; (**b**) FBG inscription by a femtosecond laser (FSL), which can be tailored to every optical wavelength.

The inscription by UV light is based on a hydrogenated photosensitive fibre, where the light is coupled through a phase mask onto the fibre core, changing the refractive index periodically (Figure 4a). In the case of the FSL, advantages over the standard UV inscription, such as the abandonment of hydrogenation and the fixed Bragg wavelength determined by the static phase mask, become possible. A highly energetic laser beam is focused into the core of the fibre and changes the refractive index by inscribing voids into the core (Figure 4b). The advanced technique allows a precise tailored Bragg wavelength coinciding with the optical FPI spectrum. Furthermore, a post-FBG inscription in an EFPI sensor was also demonstrated recently by our research group [21], which enables the improvement of existing FPI-based pressure sensors in terms of manufacturability.

### 3.2. Fabry–Perot Interferometer Pressure Sensor

**Figure 5:**
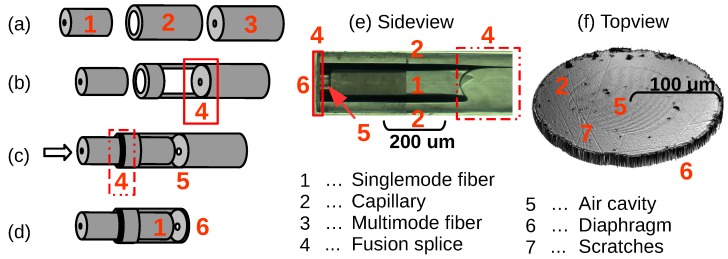
The optical fibre pressure and temperature sensor assembly steps: (**a**) SMF, glass capillary (CAP) and multi-mode fibre (MMF); (**b**) the CAP is spliced to the MMF; (**c**) and the SMF is pushed into the CAP and fused together; (**d**) the MMF is polished to become the diaphragm; (**e**) 400× side view magnification of the OFPTS; (**f**) top view of the diaphragm, captured by a Vecco interferometer.

To form the FPI structure of the sensor in this investigation, a multi-mode fibre (MMF), with 200 *μ*m diameter, was fused to a fusion splicer at the end of a glass-capillary with an inner diameter of 130 *μ*m and outer diameter of 200 *μ*m (Figure 5a,b). To ensure a high quality, the MMF and the glass capillary (CAP) were polished with 0.3 *μ*m diamond polish paper, checked by a 400× microscope and cleaned by an ultrasonic bath filled with isopropyl alcohol (IPA). A Siemens Corning M90 fusion splicer was used to fuse the all-glass components together and to completely seal the junction, which is essential to guarantee the long-term stability of the OFS. Afterwards the SMF (125 *μ*m diameter), with the FBG located close to the tip, was pushed into the CAP, while the reflected spectrum is continuously monitored. The SMF end-face with the MMF forms a 20–30 *μ*m air-filled cavity (Figure 5c), estimated from the measured spectrum. The FBG is located close to the EFPI to guarantee an approximately close spatial pressure and temperature measurement. A fixed and completely sealed FPI was achieved by fusing the CAP to the SMF. The FBG has a reflectivity of ∼99%, which is high compared to the reflectivity of an FPI (∼4%). During a 10-s fusion process with 12 mA, a temperature of ($∼650{\;}^{\circ }$C) was achieved necessary for the CAP to collapse and merge with the SMF. During the fusion process, the reflectivity of the FBG decreases to ∼1%–10%.

**Figure 6:**
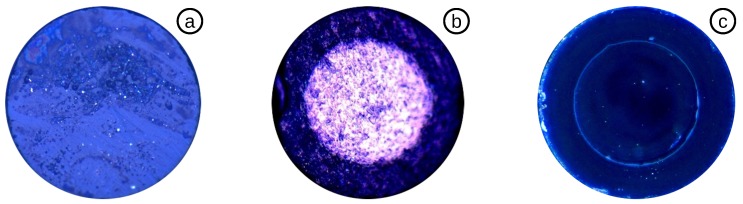
Top view of: (**a**) cut MMF without polishing; (**b**) MMF polished down to 12–20 *μ*m; (**c**) MMF polished down to 4–8 *μ*m, captured by a Zeiss microscope.

The MMF was cleaved 50–400 *μ*m away from the CAP-MMF fuse by a diamond blade (Figure 5d), creating a relatively rough surface (Figure 6a). The sensor was subsequently clamped in a subminiature Version A (SMA) holder and polished on diamond polish paper with a grit size of 3 *μ*m to achieve a thickness of 12–20 *μ*m (Figure 6b). A low-sensitivity pressure sensor was achieved by polishing the diaphragm, on a 0.3 *μ*m diamond polish paper, to a thickness of 4–8 *μ*m. The top-view of the sensor, shown in Figure 6c, indicates the clean diaphragm surface without any scratches and holes. To guarantee a clean surface, the whole polishing process is checked visually using a 400× inspection microscope, and the spectrum is continuously measured online. For high-pressure systems (e.g., deep ocean measurements), this sensor is complete at this stage.

**Figure 7:**
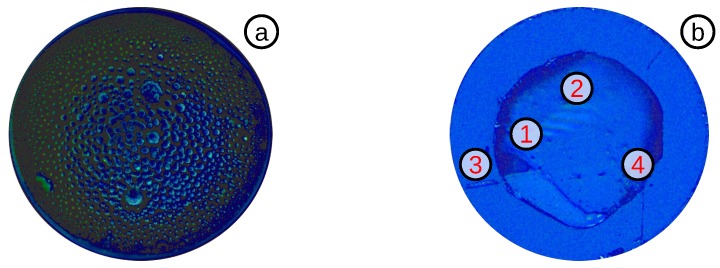
(**a**) Glass particles inside of the cavity; (**b**) broken diaphragm caused by over-polishing with: (1) a hole; (2) glass inside of the cavity; (3,4) deep scratches.

Figure 7a is focused on the inside of the EFPI cavity, where glass particles formed during the vibration of the polish process, caused by a poorly-cleaned capillary. The pressure sensitivity is inversely proportional to the third power of the diaphragm thickness, as shown in Equation (2). For high-sensitivity biomedical applications, a diaphragm with a 65-*μ*m radius requires a thickness of 2–3 *μ*m. However, a glass diaphragm below 4 *μ*m is too fragile for polishing and tends to break very easily. A hole caused by continued polishing below a diaphragm thickness of 4 *μ*m is shown in Figure 7b.

### 3.3. Etching the Diaphragm High-Sensitivity OFPTS

Initially, a Zeiss microscope with 400× magnification measured the precise thickness of the diaphragm. To achieve high pressure sensitivity, the diaphragm has to be etched from 4–8 *μ*m to 2–3 *μ*m. This can be achieved using a focused ion beam (FIB), hydrofluoric (HF) acid or an FSL. During the etching process, the diaphragm thickness reduces continuously until it reaches the critical point of high sensitivity and low stability.

#### 3.3.1. Diaphragm Reduction by HF Acid

**Figure 8:**
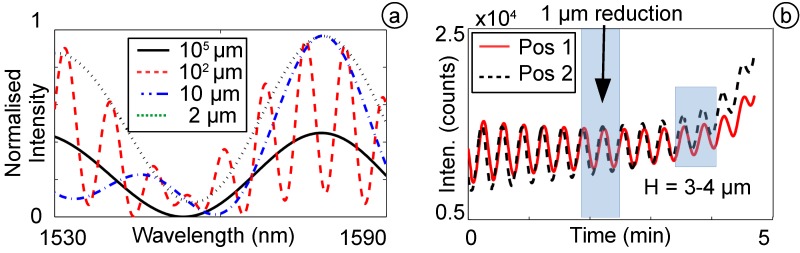
(**a**) FPI spectrum for different diaphragm thicknesses; (**b**) change of intensity during the etching process.

A continuous isotropic etching process in 48 % concentration of HF acid requires special safety requirements, such as a fume hood and safety clothes. The etching process was controlled online and the optical spectrum of the sensor analysed continuously (Figure 8a). During the etching, the diaphragm thickness reduced continuously, allowing for precise estimation of the diaphragm thickness using online monitoring techniques. The online monitoring technique comprises a dynamic tracking algorithm and estimates the diaphragm thickness in real time, regardless of the etching rate [12]. Since the reduction of the diaphragm thickness changes the spectrum (e.g., at the Q-point of the FPI signal) periodically, the etching results in a periodical oscillation in intensity. One period in intensity oscillation of the spectrum represents ∼0.5 *μ*m of thickness reduction. Additionally, the third cosine term in Equation (1) is broadening in its period, resulting in an increased separation of two selected Q-Points in the FPI signal, which can be observed when the diaphragm thickness is approximately *h* = 3–4 *μ*m (Figure 8b).

During the fusion process of SMF and CAP, the sensor was heated for 10 s before the CAP collapsed and sealed the sensor completely. This low current and long time fusion splice was chosen to increase the volume of the air to achieve a low pressure inside the cavity after the cooling phase, which assists the signal analysis during the etching process. When the diaphragm reaches a thickness of ∼3–4 *μ*m, it starts to bend, resulting from the pressure difference to the outer medium. This is measured during the etching process and used to estimate the diaphragm thickness. This method is accurate enough to guarantee a high sensitivity OFPTS fabrication success rate of better than 80%. After the etching process, the sensor is cleaned carefully for more than one hour in a water bath to remove any residual HF particles.

#### 3.3.2. Diaphragm Reduction by a FSL

HF acid etching techniques have two major drawbacks. Firstly, isotropic etching acts on the whole fibre structure, reducing the diaphragm thickness, but also the wall thickness of the capillary. This reduces the mechanical integrity of the whole structure. Secondly if insufficient care is taken during the fabrication process, residual HF can penetrate within the sensor structure and continue etching, weakening the optical structure and impairing the long-term stability of the sensor by micro-leakages.

**Figure 9:**
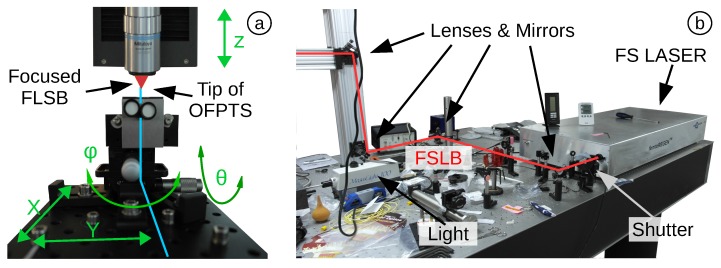
(**a**) OFPTS mounted vertically on the 3D-stage system; (**b**) FSL HighQ femtoREGENT 355 and equipment.

An FSL offers an alternative method to reduce the diaphragm thickness. The OFPTS is adjusted on a 3D-stage system (Aerotech ABL1000) with nanometre precision controlled by a computer. The stage holds the sensor with the tip pointing towards the focus lens (Figure 9a). The tilt of the holder is manually adjustable in two directions (*ϕ* and *θ*) to allow positioning the diaphragm to the focal point. The etching was undertaken using an FSL HighQ femtoREGEN 355 (Figure 9b). The pulse energy is adjustable up to 10μJ, with a pulse width of 300 fs at a wavelength of 1035 nm ± 5 nm. To guarantee an equally spatially-distributed femtosecond laser beam (FSLB), lenses and mirrors guided the laser pulse to a high level of precision when focused on the diaphragm. A specially-designed code was loaded into the computer to move the stage and to control the FSL.

**Figure 10:**
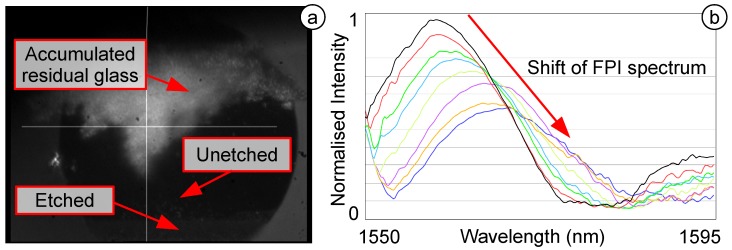
(**a**) Etching process by the FSL; (**b**) reflected FPI spectrum.

During the etching process, an increased amount of residual particles was observed, leading to an increased thickness in the remaining area (Figure 10a). During the etching process, the FPI spectrum was acquired also online and demonstrated a shift during the etching (Figure 10b). This was observed to be similar to the etching with HF acid, indicating a successful etching step. As soon as the FSLB etches the area of the diaphragm where the core of the SMF reflects the light, the reflective signal was extinguished. This can be explained by the ridge structure of the diaphragm, causing a scattering of the light. This initial results indicate that this highly novel technique works, but more work is required to improve the method.

#### 3.3.3. Highly Sensitive OFPTS

**Figure 11:**
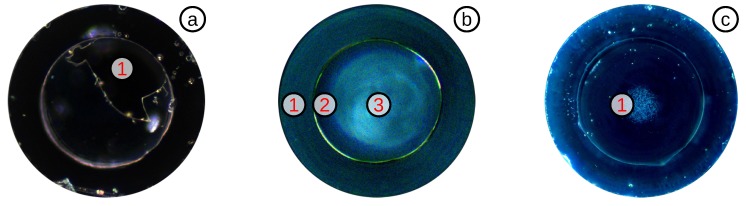
Top-view of: (**a**) a diaphragm with a big hole (1) caused by over-etching; (**b**) clean 2–3 *μ*m-thick diaphragm with: (1) CAP; (2) cavity and (3) SMF; (**c**) long-term used sensor, with small accumulated glass particles (1).

As the OFPTS diaphragm becomes thinner, it will be also more fragile. If the diaphragm becomes too thin, it can crack during the etching (Figure 11a). If the etching process was successful, the sensor tip appears clean, and the SMF becomes clearly visible (Figure 11b). After using the sensor extensively for measurements, small glass particles can break from the walls and accumulate on the inside of the diaphragm (Figure 11c). To avoid this accumulation, a clean environment and fabrication process is mandatory.

## 4. Testing and Results of the OFPTS System

The OFPTS were cleaned and tested for sensitivity, resolution and stability in both pressure and temperature. These experimental tests were conducted in water and in air.

### 4.1. Pressure Sensitivity and Resolution

**Figure 12:**
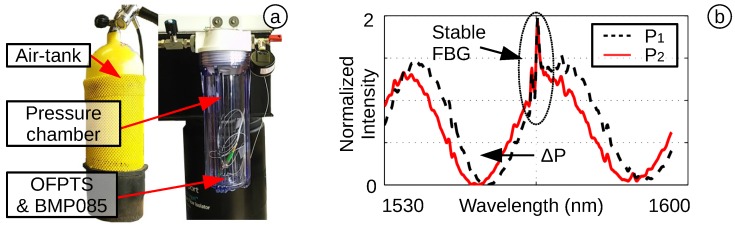
(**a**) Pressure chamber up to 10 bar; (**b**) FPI spectrum with stable FBG.

The OFPTS are first tested for their pressure sensitivity ($s_{P}$). The shift of the FPI spectrum is calculated by applying a pressure up to 100 kPa, in a pressure chamber designed for the OFPTS (Figure 12a). The OFPTS is mounted in the chamber to a ferrule connector (FC) and is in close proximity to an Arduino nano Board with a BMP085 pressure reference sensor. An air-tank pumped compressed air into the chamber, which is capable of containing pressure up to 10 bar. This caused a shift in the FPI spectrum (Figure 12b). The fixed FBG indicates a stable temperature in the chamber. The pressure sensitivity ($s_{p}$) was calculated by the initial position and the position at the end pressure level, corresponding to Equation (2), and stored in the computer for each sensor individually.

**Figure 13:**
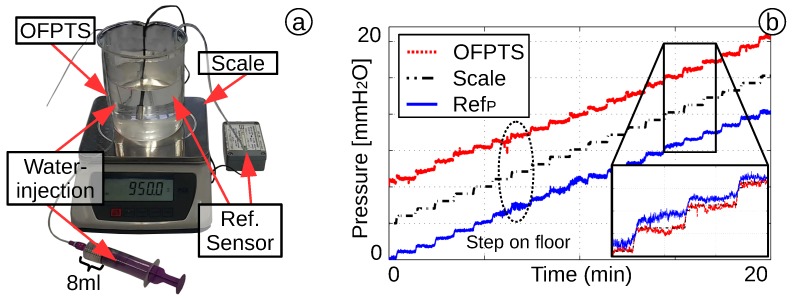
(**a**) Beaker with OFPTS, MPXV7002 reference sensor and scale; (**b**) increased pressure with successive 1-mm steps of water.

The OFPTS present in this investigation has achieved a sensitivity range of 2–10 $\frac{\text{nm}}{\text{kPa}}$, depending on the diaphragm thickness. This allows a pressure resolution of better than 0.1 cm H${}_{2}$O. To analyse the pressure resolution, the sensor is mounted in a beaker filled with water. A reference sensor (Freescale Semiconductor, MPXV7002) with a pressure range of -2–+2 kPa was also mounted on the beaker. The water level was increased in 1-mm steps every 1 min, with a syringe of 8 mL of water to minimize interference. Additionally, a scale was used to calculate the water level by their weight (Figure 13a). The result (Figure 13b) demonstrates the good correlations of the OFPTS to the scale and appears to be more accurate than the reference sensor. The resolution is better than ΔP = 10 Pa (0.1 cm H${}_{2}$O), which is sufficient for standard medical equipment.

### 4.2. Temperature Sensitivity and Resolution

**Figure 14:**
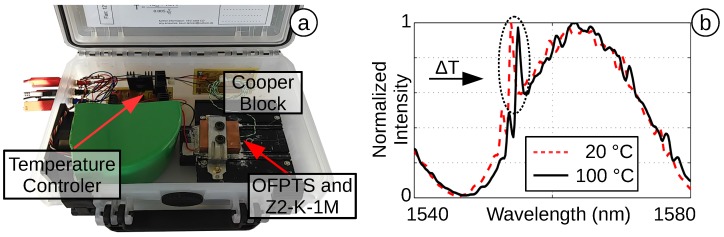
(**a**) Controlled temperature block; (**b**) FBG temperature shift.

The temperature was calculated by the spectral FBG shift, in a controlled temperature block (Figure 14a) with a Peltier element and copper block designed for the OFPTS. The copper block was mounted on a Peltier, whose temperature is controlled by a circuit connected to a data acquisition card (DAQ) card and a LabVIEW${}^{TM}$ program. The OFPTS was placed in a 0.3-mm drilled hole in the centre of the copper block. The reference sensor (k-type thermocouple, Z2-K-1M) was placed in a hole directly underneath the OFPTS hole. To guarantee a stable thermal environment, the whole block is covered by insulation material. The system increase the temperature up to $100{\;}^{\circ }$C to analyse the FBG shift (Figure 14b). The measurement shows a temperature sensitivity of k∼10.7$\frac{\text{pm}}{\text{K}}$ with a pre-inscribed FBG and with the FSL post-inscription.

**Figure 15:**
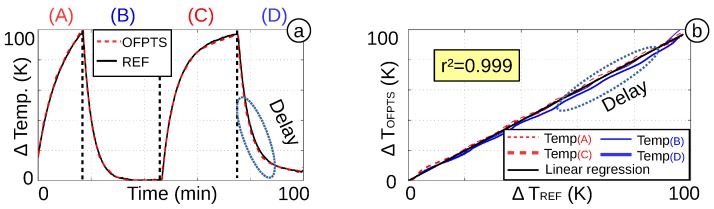
(**a**) One hundred minutes with two heating and cooling phases; (**b**) temperature linearity.

To guarantee a repeatable temperature measurement, the sensor is heated and cooled down repeatedly with a temperature difference of ΔT=100 K. A 100-min test shows the good correlation of the OFPTS and the reference sensor (Figure 15a). The repeated measurement (Figure 15b) shows the high linearity with $r^{2}=0.999$. The offset in the last cooling phase (D) is caused by a small delay in the electrical sensor (caused by the designed LabVIEW program). The good sensitivity combined with the adapted algorithm results in a temperature resolution of better than ΔT=0.1 K. This is sufficient to distinguish the healthy or increased temperature of a patient.

### 4.3. Short and Long Time Pressure Stability

**Figure 16:**
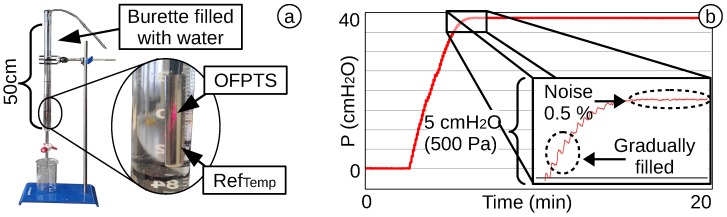
(**a**) A 50-cm burette filled with water; (**b**) 20-min stability test of an OFPTS.

To test the stability of the OFPTS, it was placed in a 50-cm burette filled with water (Figure 16a). The water was filled in successively to a level of 38 cm, and the sensor was tested afterwards for 20 min for drift and noise. The noise level is less than 0.5 % of the full scale (Figure 16b).

**Figure 17:**
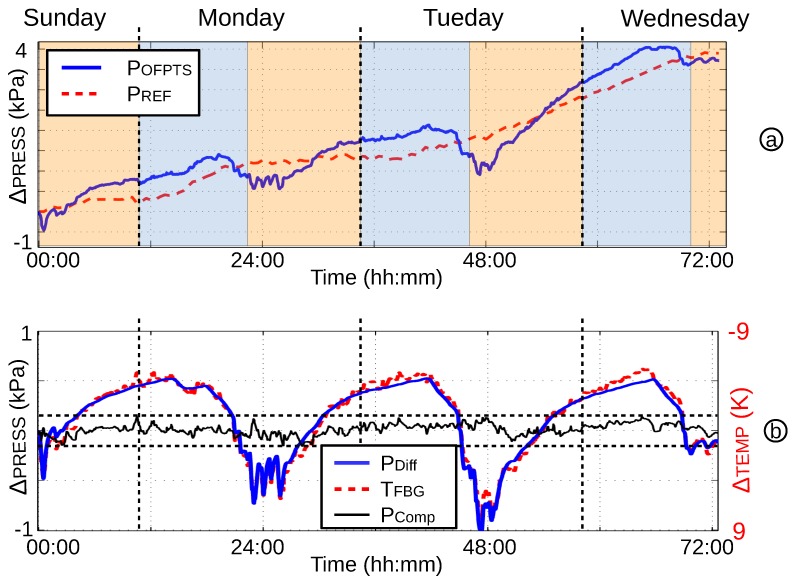
(**a**) A 72-h test with increased air pressure; (**b**) stability with compensated temperature.

After some time, the water temperature is changing, and the weather-dependent air pressure also has an affect on the pressure measurement, therefore the apparent stability. To examine the long-term stability, a continuous test with a frame rate of 10 Hz over a six-day period was undertaken. The water burette was placed near the window, where the temperature changed by ΔT≈10–15 K per day, over the whole time period. Additionally, the air pressure changed by ΔP≈4 kPa, changing the pressure measurement significantly. To demonstrate the high stability, an outline of 72 h is shown in Figure 17. The air pressure ($P_{REF}$) compared to the measured pressure ($P_{OFPTS}$) is shown in Figure 17a. The pressure increase over 4 kPa during a three-day period. The differences of both pressures are shown in Figure 17b ($P_{Diff}$) with the same trend as the temperature ($T_{FBG}$). With a compensation of the temperature, the sensor shows no observable drift in 72h of continuous online measurement. Furthermore, the difference from maximum to minimum pressure is less than 0.3 kPa (2.25 mmHg) in this 72 h.

### 4.4. Dynamic Pressure Response and Stability of the OFPTS

**Figure 18:**
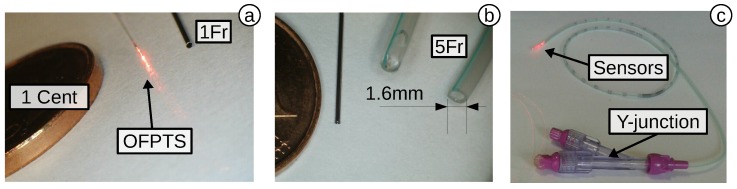
Compared to a cent: (**a**) OFPTS (red illuminated) and 1-Fr tube; (**b**) 5-Fr NutriSafe catheter; (**c**) two sensors in a single 5-Fr catheter.

The dynamic response and pressure stability are important factors for biomedical applications; particularly for *in vivo* applications, it is often extremely difficult or even impossible to recalibrate a sensor. For this reason, a 12-h measurement in an artificial heart simulator (Mecora CoroSim) filled with water was undertaken. The sensor (Figure 18a) was placed, together with a reference sensor (Opsens OPP-M), in a 1.6 mm (5 Fr)-sized NutriSafe catheter from Vygon (Figure 18b). The standard catheter, which is also used in urodynamics, has an o.d. of 1.6 mm (5 Fr) and can therefore house multiple sensors. The sensors were sealed on the tip of the NutriSafe closing cap, which also allows an additional Y-junction (Figure 18c) for flushing the catheter with water. The catheter was then guided through a 6-Fr access in the direction of the artificial heart.

**Figure 19:**
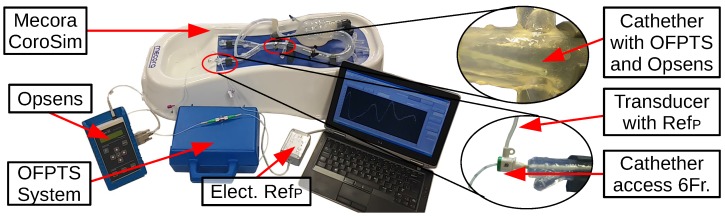
Mecoras CoroSim with Opsens and OMRON reference sensor.

The CoroSim is pulsating continuously with more than $1\frac{\text{beats}}{\text{s}}$, resulting in more than 50,000 pulses with a range of −2.7 kPa (−20 mmHg) to +8 kPa (+60 mmHg). An electrical sensor (OMRON, 2SMPP-03 MEMS Gauge) with a range of −50 kPa to 50 kPa was also connected (Figure 19).

**Figure 20:**
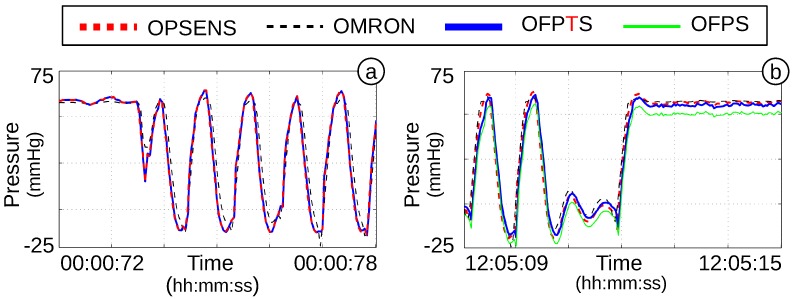
(**a**) Pressure in the first minute; (**b**) pressure after 12 h of continuous measurement.

In the beginning of the dynamic 12-h measurement, the OFPTS and the Opsens showed a very good correlation (Figure 20a). The electrical sensors showed a damping, which is caused by a different location. Both optical sensors are placed approximately close to each other in the same miniaturized catheter, whereas the electrical sensor is connected to a transducer at the inlet of the catheter access. After 12 h, all sensors still demonstrated a good correlation to each other (Figure 20b). The temperature of the water increased, caused by the continuous pressure, which caused a shift of 0.4 kPa (3 mmHg) in the OFPTS. With the aforementioned temperature compensation, the difference of OFPTS, Opsens and OMRON pressure sensors is less than 0.13 kPa (1 mmHg). This results in a change of ∼10 $\frac{\text{Pa}}{\text{h}}$, which is sufficient for biomedical applications.

## 5. Conclusions

A medical evaluation system for an optical fibre pressure and temperature sensors (OFPTS) has been realized by a combination of inexpensive hardware, with highly sensitive optical fibre sensors and advanced adapted software algorithms. The performance of all components depends partially on the sensitivity and stability of the optical fibre sensor. The sensor described in this paper is an all-glass construction based on EFPI with a flexible diaphragm located at the tip of an optical fibre. The small diameter of 200 *μ*m allows the sensor to be comfortably handled, even in volume-restricted areas.

A continuous development of the sensing and fabrication technique is necessary for high standard medical application, especially for small-sized diaphragm-based sensors. A comparison of different diaphragm shrinking techniques based on polishing, HF acid and an FS laser were undertaken. The results demonstrated the borders of polishing and the necessity for use of an advance etching technique. HF acid results in a good performance during etching; however, it also shows that a non-cleaned sensor can be effected long after fabrication. The technique based on an FSL demonstrated the possibility of a safer etching process, but more work is required to establish the efficiency of this method. The HF acid fabrication method results in a highly sensitive sensor with a pressure sensitivity in the range of ∼2–10 $\frac{\text{nm}}{\text{kPa}}$, no drift in 72 h and a resolution of better than 10 Pa (0.1 cmH${}_{2}$O).

The sensor is also capable of simultaneous pressure and temperature measurement. The sensor design is based on the combination of the selection of an EFPI with a closely-located integrated FBG. Furthermore, the temperature measurement of the FBG can also compensate for any FPI drift due to temperature variations resulting in a low-drift pressure sensor. The OFPTS were fabricated with pre-inscribed FBG fibres (UV-inscription and draw-tower) and with a FBG post-inscription technique (inscribed by a FSL). Both techniques demonstrated good performance with a temperature resolution of better than 0.1 K and a high repeatability with a correlation factor of $r^{2}=0.999$.
